# A Networked Meta-Population Epidemic Model with Population Flow and Its Application to the Prediction of the COVID-19 Pandemic

**DOI:** 10.3390/e26080654

**Published:** 2024-07-30

**Authors:** Dong Xue, Naichao Liu, Xinyi Chen, Fangzhou Liu

**Affiliations:** 1 Key Laboratory of Smart Manufacturing in Energy Chemical Process, Ministry of Education, East China University of Science and Technology, Shanghai 200237, China; 2 Research Institute of Intelligent Control and Systems, School of Astronautics, Harbin Institute of Technology, Harbin 150001, China

**Keywords:** epidemics model, discrete-time networked SAIR model, population flow, joint parameter-topology estimation

## Abstract

This article addresses the crucial issues of how asymptomatic individuals and population movements influence the spread of epidemics. Specifically, a discrete-time networked Susceptible-Asymptomatic-Infected-Recovered (SAIR) model that integrates population flow is introduced to investigate the dynamics of epidemic transmission among individuals. In contrast to existing data-driven system identification approaches that identify the network structure or system parameters separately, a joint estimation framework is developed in this study. The joint framework incorporates historical measurements and enables the simultaneous estimation of transmission topology and epidemic factors. The use of the joint estimation scheme reduces the estimation error. The stability of equilibria and convergence behaviors of proposed dynamics are then analyzed. Furthermore, the sensitivity of the proposed model to population movements is evaluated in terms of the basic reproduction number. This article also rigorously investigates the effectiveness of non-pharmaceutical interventions via distributively controlling population flow in curbing virus transmission. It is found that the population flow control strategy reduces the number of infections during the epidemic.

## 1. Introduction

The coronavirus disease 2019 (COVID-19) has been declared a pandemic by the World Health Organization [1]. The COVID-19 pandemic, resulting from severe acute respiratory syndrome coronavirus 2 (SARS-CoV-2), has become one of the deadliest epidemics in human history. Up to April 2024, there were over 775 million confirmed cases and more than 7 million reported deaths. The far-reaching impact of COVID-19 has disrupted the world economy and posed a substantial threat to public health [2]. To reduce the adverse impact of the epidemic, comprehensive tracking of the spread of the virus, timely identification and tracing of patients, and public health measures are needed.

The mathematical modeling of infectious diseases is a fundamental research area in epidemiology and public health [3,4]. In this field, compartmental models serve as essential tools for studying the transmission dynamics of epidemics, with Susceptible (S) and Infected (I) being the most common compartments [5]. Among these models, the Susceptible-Infected-Susceptible (SIS) and Susceptible-Infected-Recovered (SIR) models are particularly foundational [6]. However, the SIS and SIR models have limitations as they do not account for factors such as exposed and asymptomatic individuals. To address these limitations, more sophisticated models have been proposed [7,8]. The Susceptible-Exposed-Infected-Recovered (SEIR) model, which includes an incubation period for individuals during the transmission of an epidemic, has become widely used [9]. Similarly, the Susceptible-Asymptomatic-Infected-Recovered (SAIR) model has been proposed to describe individuals who exhibit no physical symptoms but remain contagious. In general, models that include both exposed and asymptomatic individuals are more capable of accurately describing the epidemic process. However, the increase in the number of compartments significantly complicates theoretical analysis and simulations. From a practical perspective, including asymptomatic patients in statistical models enhances prediction and analysis. Therefore, after careful consideration, we have adopted the SAIR model. It is important to note that compartmental models are based on several simplifying assumptions, such as uniform infection and recovery rates for all individuals, a well-mixed population, and a relatively small population size. These assumptions, while necessary for tractability, introduce certain limitations to the model’s applicability [10].

Global connectivity has proven to be a key factor in the spread of infectious diseases [11]. Given the impact of population movements on disease transmission, accurate modeling of such movements is crucial. Therefore, the networked model is introduced. Traditional compartmental models usually assume that the contacts between individuals are evenly mixed, while the contact patterns in reality are generally heterogeneous [12]. The networked model can account for the states of all individuals independently and reflect the contact network between individuals more accurately. A networked SIS model incorporating population mobility is proposed in [13]. The authors extended the networked SIS model and provided stability conditions for the disease equilibrium state. They also investigated the impact of individual mobility on system convergence, ensuring that the model accurately captures the complex dynamics of epidemic transmission. The networked SIS model provides comprehensive insights into population movements, but its simplified compartmental representation has inherent limitations. Consequently, researchers have introduced discrete-time networked SIR and SEIR models and analyzed their limiting behaviors [14]. The authors introduced discrete-time networked SIR and SEIR models, analyzed the limiting behavior of the models, and proposed a method to estimate propagation parameters from data. The rationality of the work was verified by simulations. Furthermore, identifying epidemic parameters and the topology structure of the networked models plays an important role in the dynamical analysis. When the topology is fixed, the epidemic parameters can be trained by a data-driven method [15]. In another work, the authors selected the values of epidemic parameters within a certain range and fitted them with the collected epidemic data to find more suitable epidemic parameter values. Then, the authors designed an objective function based on the known parameters to solve the topology structure [16]. However, the epidemic parameters and topology during the transmission process are often unknown. Hence, it is imperative to integrate data to jointly identify epidemic parameters and topology.

This article conducts a study on epidemic transmission using a networked meta-population model that incorporates population flow. The article focuses on the impact of asymptomatic individuals on the spread of the epidemic and, to simplify the modeling task, excludes the consideration of the exposed state. Consequently, a SAIR model is proposed to investigate the spread of epidemics among populations. Then, given the convenience and frequency of human travel in the era of globalization, the impact of population mobility on the spread of epidemics cannot be ignored. Therefore, the SAIR model integrates population movements, represented by the mobility rate of each state. In the subsequent analysis, the properties of the proposed model are examined to ensure its validity. Moreover, this article proposes a joint estimation scheme for epidemic parameters and network topology, compared to existing data-driven training methods. Finally, the influence of control policies on epidemic spread is explored.

The article is structured as follows. Section 2 introduces mathematical notation and graph theory. The derivation of the model is also presented in this section. In Section 3, the properties of the model are analyzed and the basic reproduction number is solved. The control strategy is also proposed. Section 4 outlines the scheme of jointly data-driven identification of epidemic parameters and network topology. Numerical simulation is conducted in Section 5. Finally, Section 6 provides the conclusion. Appendix A includes all proofs.

## 2. Preliminaries and Problem Statement

In this section, we recall some necessary notions and graph theory, introduce the networked meta-population SAIR model, and formulate the problem.

### 2.1. Notations

The sets of integers and real numbers are denoted as Z, R, respectively. For any $n\in Z_{>0}$, this article uses [n] to denote the index set {1,2,…,n}. A diagonal matrix is indicated by diag(·). The sum of the diagonal elements of a matrix is denoted as trace(·). Given a column vector x, the transpose is denoted as $x^{Τ}$. Given vectors 1 and 0, it represents the vectors whose entries are all 1 and 0. In addition, we use I to denote the identity matrix. The parameter $k\in Z_{\geq 0}$ represents a given time step. Let G=(V,E,W) denote a weighted directed graph, where V is the set of nodes and E is the set of edges, E⊆V×V. Nodes represent sub-populations and edges represent interactions between sub-populations. The weighted adjacency matrix associated with G is denotes as $W\in R^{n\times n}$, where ${\left[W\right]}_{ij}=w_{ij}$ for i,j∈[n]. The edge from node *j* to node *i* is denoted as (j,i). Parameter $w_{ij}$ represents the weight of edge (j,i), which refers to the interaction strength between sub-populations. If (j,i)∈E, one has $w_{ij}>0$, where $w_{ij}=0$ if and only if (j,i)∉E.

### 2.2. Modeling and Interpretation

In the era of globalization, the increasing ease of transportation brings both advantages for people and expedites the transmission of epidemics. Against the backdrop of the ongoing COVID-19 pandemic, there has been a notable increase in the proportion of asymptomatic patients. In addition, asymptomatic cases occur widely during the spread of certain viruses. Thus, inspired by the literature [13,17], our goal is to construct a discrete-time networked SAIR model that combines the population flow.

Specifically, a SAIR model is used to describe how susceptible individuals become asymptomatic, infected, and recovered, in which the states $S_{i},\;A_{i},\;I_{i}$, and $R_{i}$ indicate the number of susceptible, asymptomatic, infected, and recovered people of each sub-population i∈n, respectively. Sub-population *i* can be represented in practice as a city. Let $N_{i}$ be the total number of sub-population *i*; the original SAIR model is given by

(1) $$\begin{array}{cc} \; & {\dot{S}}_{i}\left(t\right)=-\beta_{i}\frac{A_{i}\left(t\right)+I_{i}\left(t\right)}{N_{i}}S_{i}\left(t\right),\\ \; & {\dot{A}}_{i}\left(t\right)=q\beta_{i}\frac{A_{i}\left(t\right)+I_{i}\left(t\right)}{N_{i}}S_{i}\left(t\right)-\sigma_{i}A_{i}\left(t\right)-\kappa_{i}A_{i}\left(t\right),\\ \; & {\dot{I}}_{i}\left(t\right)=\left(1-q\right)\beta_{i}\frac{A_{i}\left(t\right)+I_{i}\left(t\right)}{N_{i}}S_{i}\left(t\right)+\sigma_{i}A_{i}\left(t\right)-\gamma_{i}I_{i}\left(t\right),\\ \; & {\dot{R}}_{i}\left(t\right)=\kappa_{i}A_{i}\left(t\right)+\gamma_{i}I_{i}\left(t\right), \end{array}$$

where $\beta_{i}$ and $\sigma_{i}$ represent the rate of susceptible individuals being infected by asymptomatic and infected-symptomatic individuals and the rate of asymptomatic individuals becoming symptomatic infected. The variables $\kappa_{i}$ and $\gamma_{i}$ represent the recovery rate for asymptomatic individuals and the recovery rate for symptomatic infected individuals, respectively. The variable q∈[0,1] represents the fraction of susceptible individuals transitioning to an asymptomatic state upon infection and is usually previously known [18].

One can appreciate that if individuals can move between different sub-populations, the movement of infected individuals between these sub-populations leads to the spread of the epidemic [13]. Within each sub-population, contact between individuals promotes the spread of the epidemic, and the movement of people between different sub-populations also increases the risk of disease transmission. We assume that individual disease progression, and population flow processes do not affect each other, that is, the population flow process and the disease progression within each individual are independent. To account for the flow of individuals between sub-populations, Model (1) is extended as follows:

(2) $$\begin{array}{cc} \; & {\dot{S}}_{i}=-\beta_{i}\frac{A_{i}+I_{i}}{N_{i}}S_{i}+\sum_{j\neq i}\left(F_{ij}\frac{S_{j}}{N_{j}}-F_{ji}\frac{S_{i}}{N_{i}}\right),\\ \; & {\dot{A}}_{i}=q\beta_{i}\frac{A_{i}+I_{i}}{N_{i}}S_{i}-\sigma_{i}A_{i}-\kappa_{i}A_{i}+\sum_{j\neq i}\left(F_{ij}\frac{A_{j}}{N_{j}}-F_{ji}\frac{A_{i}}{N_{i}}\right),\\ \; & {\dot{I}}_{i}=\left(1-q\right)\beta_{i}\frac{A_{i}+I_{i}}{N_{i}}S_{i}+\sigma_{i}A_{i}-\gamma_{i}I_{i}+\sum_{j\neq i}\left(F_{ij}\frac{I_{j}}{N_{j}}-F_{ji}\frac{I_{i}}{N_{i}}\right),\\ \; & {\dot{R}}_{i}=\kappa_{i}A_{i}+\gamma_{i}I_{i}+\sum_{j\neq i}\left(F_{ij}\frac{R_{j}}{N_{j}}-F_{ji}\frac{R_{i}}{N_{i}}\right), \end{array}$$

where $F_{ij}$ denotes the population flow from sub-population *j* to *i*. The following assumption is proposed in order for Model (2) to be well-defined.

**Assumption** **1.** *For all i∈[n], the value of $N_{i}$ is a constant.*

Generally speaking, birth and death rates have a negligible impact on the total population over an insignificant period. Thus, Assumption 1 assumes that Model (2) is a closed system.

With the notations $s_{i}=\frac{S_{i}}{N_{i}},\;a_{i}=\frac{A_{i}}{N_{i}},\;x_{i}=\frac{I_{i}}{N_{i}},\;r_{i}=\frac{R_{i}}{N_{i}}$, the SAIR model (2) integrated the population flow turns into

(3) $$\begin{array}{cc} \; & {\dot{s}}_{i}=-\beta_{i}\left(a_{i}+x_{i}\right)s_{i}+\frac{1}{N_{i}}\sum_{j\neq i}\left(F_{ij}s_{j}-F_{ji}s_{i}\right),\\ \; & {\dot{a}}_{i}=q\beta_{i}\left(a_{i}+x_{i}\right)s_{i}-\sigma_{i}a_{i}-\kappa_{i}a_{i}+\frac{1}{N_{i}}\sum_{j\neq i}\left(F_{ij}a_{j}-F_{ji}a_{i}\right),\\ \; & {\dot{x}}_{i}=\left(1-q\right)\beta_{i}\left(a_{i}+x_{i}\right)s_{i}+\sigma_{i}a_{i}-\gamma_{i}x_{i}+\frac{1}{N_{i}}\sum_{j\neq i}\left(F_{ij}x_{j}-F_{ji}x_{i}\right),\\ \; & {\dot{r}}_{i}=\kappa_{i}a_{i}+\gamma_{i}x_{i}+\frac{1}{N_{i}}\sum_{j\neq i}\left(F_{ij}r_{j}-F_{ji}r_{i}\right), \end{array}$$

where $s_{i},\;a_{i},\;x_{i}$, and $r_{i}$ refer to the proportion of susceptible, asymptomatic, infected, and recovered individuals, respectively. The parameter $\mu_{i}$ denotes the population mobility rate of sub-population *i*. That is, $\mu_{i}$ represents the proportion of individuals’ outflow from sub-population *i*. The value of $w_{ij}$ is the proportion of individuals flowing from sub-population *j* to *i*. Using the fact that $F_{ij}=\mu_{j}w_{ij}N_{j}$ and $\sum_{j\neq i}w_{ij}=1$, the dynamics for the susceptible proportion at sub-population *i* is derived as

$$\begin{array}{cc} {\dot{s}}_{i} & =-\beta_{i}\left(a_{i}+x_{i}\right)s_{i}+\frac{1}{N_{i}}\sum_{j\neq i}\left(F_{ij}s_{j}-F_{ji}s_{i}\right)\\ & =-\beta_{i}\left(a_{i}+x_{i}\right)s_{i}+\frac{1}{N_{i}}\sum_{j\neq i}\left(\mu_{j}w_{ij}s_{j}N_{j}-\mu_{i}w_{ji}s_{i}N_{i}\right)\\ \; & =-\beta_{i}\left(a_{i}+x_{i}\right)s_{i}-\mu_{i}s_{i}+\frac{1}{N_{i}}\sum_{j\neq i}\mu_{j}w_{ij}s_{j}N_{j}. \end{array}$$

In a spirit similar to the above derivations, Model (3) can be rewritten as

(4) $$\begin{array}{cc} \; & {\dot{s}}_{i}=-\beta_{i}\left(a_{i}+x_{i}\right)s_{i}-\mu_{i}s_{i}+\frac{1}{N_{i}}\sum_{j\neq i}\mu_{j}w_{ij}s_{j}N_{j},\\ \; & {\dot{a}}_{i}=q\beta_{i}\left(a_{i}+x_{i}\right)s_{i}-\left(\sigma_{i}+\kappa_{i}+\mu_{i}\right)a_{i}+\frac{1}{N_{i}}\sum_{j\neq i}\mu_{j}w_{ij}a_{j}N_{j},\\ \; & {\dot{x}}_{i}=\left(1-q\right)\beta_{i}\left(a_{i}+x_{i}\right)s_{i}-\left(\gamma_{i}+\mu_{i}\right)x_{i}+\sigma_{i}a_{i}+\frac{1}{N_{i}}\sum_{j\neq i}\mu_{j}w_{ij}x_{j}N_{j},\\ \; & {\dot{r}}_{i}=\kappa_{i}a_{i}+\gamma_{i}x_{i}-\mu_{i}r_{i}+\frac{1}{N_{i}}\sum_{j\neq i}\mu_{j}w_{ij}r_{j}N_{j}. \end{array}$$

The model is then discretized given the nature of the collected data on the pandemic spread, where the highest resolution data are typically recorded once per day. As a result, the discrete-time counterpart of the continuous-time model (4) can be written as follows

(5) $$\begin{array}{cc} s_{i}^{k+1}= & s_{i}^{k}+h\left(-\beta_{i}\left(a_{i}^{k}+x_{i}^{k}\right)s_{i}^{k}-\mu_{i}s_{i}^{k}+\frac{1}{N_{i}}\sum_{j\neq i}\mu_{j}w_{ij}s_{j}^{k}N_{j}\right),\\ a_{i}^{k+1}= & a_{i}^{k}+h\left(q\beta_{i}\left(a_{i}^{k}+x_{i}^{k}\right)s_{i}^{k}-\left(\sigma_{i}+\kappa_{i}+\mu_{i}\right)a_{i}^{k}+\frac{1}{N_{i}}\sum_{j\neq i}\mu_{j}w_{ij}a_{j}^{k}N_{j}\right),\\ x_{i}^{k+1}= & x_{i}^{k}+h\left(\left(1-q\right)\beta_{i}\left(a_{i}^{k}+x_{i}^{k}\right)s_{i}^{k}-\left(\gamma_{i}+\mu_{i}\right)x_{i}^{k}+\sigma_{i}a_{i}^{k}+\frac{1}{N_{i}}\sum_{j\neq i}\mu_{j}w_{ij}x_{j}^{k}N_{j}\right),\\ r_{i}^{k+1}= & r_{i}^{k}+h\left(\kappa_{i}a_{i}^{k}+\gamma_{i}x_{i}^{k}-\mu_{i}r_{i}^{k}+\frac{1}{N_{i}}\sum_{j\neq i}\mu_{j}w_{ij}r_{j}^{k}N_{j}\right), \end{array}$$

where $s_{i}^{k}$, $a_{i}^{k}$, $x_{i}^{k}$, and $r_{i}^{k}$ represent the proportion of susceptible, asymptomatic, infected, and recovered individuals in sub-population *i* at time *k*. Parameter $h\in R_{>0}$ represents the sampling interval. Consequently, a networked meta-population SAIR model with population flow is presented.

## 3. Analysis and Control of the Model

The properties of Model (5) are analyzed in this section to illustrate its applicability. Moreover, an expression for the basic reproduction number is derived. A control strategy is also proposed to simulate the effect of control policies on epidemic transmission.

### 3.1. Stability and Convergence Analysis

The stability and convergence analysis of the networked SAIR model with population flow is presented in this subsection. First, the following assumption is necessary to guarantee that the proposed Model (5) is well-defined.

**Assumption** **2.** *For all i,j∈[n], one has $0<h\mu_{i}<1$, $0<h\beta_{i}<1$, $0<h\sigma_{i}<1$, $0<h\gamma_{i}<1$, $0<h\kappa_{i}<1$, $0<h(\beta_{i}+\mu_{i})<1$, $0<h(\gamma_{i}+\mu_{i})<1$, $0<h(\sigma_{i}+\kappa_{i}+\mu_{i})<1$ and $0<h\sum_{j\neq i}\mu_{j}w_{ij}<1$.*

Concerning the epidemic parameters, Assumption 2 necessitates that the sampling interval, *h*, remains sufficiently small.

**Proposition** **1.** *Consider Model (5) under Assumptions 1 and 2; suppose $s_{i}^{0},a_{i}^{0},x_{i}^{0},r_{i}^{0}\in \left[0,1\right]$ and $s_{i}^{0}+a_{i}^{0}+x_{i}^{0}+r_{i}^{0}=1$ for all i∈[n]. Then, when k≥0, there exists $s_{i}^{k},a_{i}^{k},x_{i}^{k},r_{i}^{k}\in \left[0,1\right]$ and $s_{i}^{k}+a_{i}^{k}+x_{i}^{k}+r_{i}^{k}=1$ for all i∈[n].*

To this end, the model is well-defined based on relevant assumptions and propositions. Next, the following lemma and theorem show the stability and convergence analysis of the proposed model.

**Lemma** **1.** Consider Model (5) under Assumptions 1 and 2. For some i∈[n], such that $a_{i}^{0},x_{i}^{0}\in \left(0,1\right]$, there exists a set of asymptotically stable equilibria of the form $\left(s^{*},a^{*},x^{*},r^{*}\right)$, where $s^{*}={\left(s_{1}^{*},s_{2}^{*},\ldots ,s_{n}^{*}\right)}^{Τ}$, $a^{*}={\left(a_{1}^{*},a_{2}^{*},\ldots ,a_{n}^{*}\right)}^{Τ}$, $x^{*}={\left(x_{1}^{*},x_{2}^{*},\ldots ,x_{n}^{*}\right)}^{Τ}$, $r^{*}={\left(r_{1}^{*},r_{2}^{*},\ldots ,r_{n}^{*}\right)}^{Τ}$, and $a^{*}=x^{*}=0$, $r^{*}=1-s^{*}$, $s^{*}\in \left[0,1\right]$.

Since the proof of Lemma 1 can be straightforwardly derived by referring to Theorem 1 in [19], it is saved for triviality. According to Lemma 1, the state $\left(s^{*},0,0,1-s^{*}\right)$ is a set of equilibria of Model (5) and thus as the model asymptotically reaches the equilibria where $a^{*}=x^{*}=0$, only susceptible and recovered individuals exist in the system.

Let $\lambda_{max}^{M_{k}}$ be the dominant eigenvalue of $M_{k}$, where $M_{k}$ is defined as

$$\begin{array}{c} M_{k}=\left[\begin{array}{cc} M_{11} & M_{12}\\ M_{21} & M_{22} \end{array}\right], \end{array}$$

where $M_{11}=I+h\left(-\left(\sigma +\kappa +\mu \right)+\beta q\mathrm{diag}\left(s_{i}^{k}\right)+N^{-1}W\mu N\right)$, $M_{12}=h\beta q\mathrm{diag}\left(s_{i}^{k}\right)$, $M_{21}=h\beta \left(1-q\right)\mathrm{diag}\left(s_{i}^{k}\right)+h\sigma$, $M_{22}=I+h\left(-\left(\gamma +\mu \right)+\beta \left(1-q\right)\mathrm{diag}\left(s_{i}^{k}\right)+N^{-1}W\mu N\right)$, $\beta =\mathrm{diag}(\beta_{i})$, $\sigma =\mathrm{diag}(\sigma_{i})$, $\gamma =\mathrm{diag}(\gamma_{i})$, $\kappa =\mathrm{diag}(\kappa_{i})$, $\mu =\mathrm{diag}(\mu_{i})$, $N=\mathrm{diag}(N_{i})$.

**Theorem** **1.** *Consider Model (5) under Assumptions 1 and 2. For some i∈[n], such that $a_{i}^{0}>0$ and $x_{i}^{0}>0$. Then, for all i∈[n],*

*(i)*  *$s_{i}^{k+1}\leq s_{i}^{k}$ for all k≥0,*
*(ii)*  *$\lim_{k\to \infty }a_{i}^{k}=x_{i}^{k}=0$,*
*(iii)*  *$\lambda_{max}^{M_{k}}$ is monotonically decreasing as a function of k,*
*(iv)*  *there exists $k_{0}$, satisfying $\lambda_{max}^{M_{k}}<1$ when $k\geq k_{0}$.*

The related proof is given in Appendix A. Theorem 1 states that the proportion of susceptible individuals declines during the transmission of epidemics, and with the development of vaccines and the improvement of immunity, the epidemics disappear eventually.

### 3.2. Basic Reproduction Number

In this subsection, we explore the basic reproduction number of the proposed epidemic model to characterize the infection state of the virus.

This article quantifies the transmissibility of viruses via basic reproduction number $R_{0}$. Some basic references point out that if $R_{0}>1$, it means the epidemic invades. If $R_{0}<1$, the epidemic gradually subsides [5,20]. In [21], the author introduces a method for solving the basic reproduction number, where the two related coefficient matrices F and V are given by

$$\begin{array}{cc} \; & F=\left[\begin{array}{cc} q\beta_{i} & q\beta_{i}\\ \left(1-q\right)\beta_{i} & \left(1-q\right)\beta_{i} \end{array}\right],\\ \; & V=\left[\begin{array}{cc} \sigma_{i}+\kappa_{i}+\mu_{i}-\theta_{i} & 0\\ -\sigma_{i} & \gamma_{i}+\mu_{i}-\theta_{i} \end{array}\right], \end{array}$$

where $\theta_{i}={N_{i}}^{-1}\sum_{j\neq i}\mu_{j}w_{ij}N_{j}$. Let $R_{0}=\rho \left({\mathrm{FV}}^{-1}\right)$ be the basic reproduction number, where ρ(·) is the spectral radius of matrix

(6) $$\begin{array}{cc} \; & {\mathrm{FV}}^{-1}=\left[\begin{array}{cc} \frac{q\beta_{i}}{\sigma_{i}+\kappa_{i}+\mu_{i}-\theta_{i}}+\frac{q\sigma_{i}\beta_{i}}{\left(\sigma_{i}+\kappa_{i}+\mu_{i}-\theta_{i}\right)\left(\gamma_{i}+\mu_{i}-\theta_{i}\right)} & \frac{q\beta_{i}}{\gamma_{i}+\mu_{i}-\theta_{i}}\\ \frac{\left(1-q\right)\beta_{i}}{\sigma_{i}+\kappa_{i}+\mu_{i}-\theta_{i}}+\frac{\left(1-q\right)\sigma_{i}\beta_{i}}{\left(\sigma_{i}+\kappa_{i}+\mu_{i}-\theta_{i}\right)\left(\gamma_{i}+\mu_{i}-\theta_{i}\right)} & \frac{\left(1-q\right)\beta_{i}}{\gamma_{i}+\mu_{i}-\theta_{i}} \end{array}\right], \end{array}$$

which has a zero eigenvalue. Thus, the spectral radius of matrix $FV^{-1}$, given in Equation (6), can be calculated in a straightforward manner as

$$\begin{array}{cc} \rho \left({\mathrm{FV}}^{-1}\right)=\mathrm{trace}\left({\mathrm{FV}}^{-1}\right)= & \frac{q\beta_{i}}{\sigma_{i}+\kappa_{i}+\mu_{i}-\theta_{i}}+\frac{\left(1-q\right)\beta_{i}}{\gamma_{i}+\mu_{i}-\theta_{i}}\\ & +\frac{q\sigma_{i}\beta_{i}}{\left(\sigma_{i}+\kappa_{i}+\mu_{i}-\theta_{i}\right)\left(\gamma_{i}+\mu_{i}-\theta_{i}\right)}, \end{array}$$

which results in

(7) $$\begin{array}{c} R_{0}=\frac{q\beta_{i}}{\sigma_{i}+\kappa_{i}+\mu_{i}-\theta_{i}}+\frac{q\sigma_{i}\beta_{i}}{\left(\sigma_{i}+\kappa_{i}+\mu_{i}-\theta_{i}\right)\left(\gamma_{i}+\mu_{i}-\theta_{i}\right)}+\frac{\left(1-q\right)\beta_{i}}{\gamma_{i}+\mu_{i}-\theta_{i}}. \end{array}$$

In analogy with Equation (7), the basic reproduction number of epidemic-spreading processes that disregard population flows can be derived as

(8) $$\begin{array}{cc} R_{0}^{\prime }=\rho \left({\mathrm{FV}}^{-1}\right) & =trace\left({\mathrm{FV}}^{-1}\right)=\frac{q\beta_{i}}{\sigma_{i}+\kappa_{i}}+\frac{q\sigma_{i}\beta_{i}}{\left(\sigma_{i}+\kappa_{i}\right)\gamma_{i}}+\frac{\left(1-q\right)\beta_{i}}{\gamma_{i}}, \end{array}$$

which shows that the basic reproduction number in the absence of population flow depends on the epidemic parameters $\beta_{i},\;\sigma_{i},\;\kappa_{i}$, and $\gamma_{i}$. As shown in Equation (7), the determination of $R_{0}$ is contingent on the epidemic parameters and the population mobility rate. A comparison of Equations (8) and (7) reveals that population movements can influence epidemic transmission.

### 3.3. Population Flow Control Strategy

Pharmaceutical interventions are important in inhibiting virus replication, but they often have certain side effects, and prolonged usage may lead to pharmaceutical resistance. Therefore, nucleic acid detection and genetic sequencing of viruses are essential for monitoring existing viral strains and identifying potential mutations. Additionally, maximizing vaccination coverage is imperative because it enhances individual immunity and reduces the risk of infection. Pharmaceutical interventions contribute to reducing the infection rate and improving the recovery rate.

Non-pharmaceutical interventions encompass measures such as stay-at-home orders, curfews, and quarantines to mitigate the risk of infection by limiting interpersonal contact. Considering the impact of population flow on disease spread, controlling the movement of people is crucial. Consequently, this study proposes a population flow control strategy to simulate the impact of different control policies on the spread of epidemics. The strategy is indicated as follows:

(9) $$\begin{array}{c} \mu_{i}=\mu_{imax}+\left(\mu_{imin}-\mu_{imax}\right)\eta , \end{array}$$

where η changes according to the strength of government control. If η=1, there is a complete lockdown. If η=0, there is no control policy. Moreover, parameters $\mu_{imax}$ and $\mu_{imin}$ are the maximum and minimum values of the population mobility rate $\mu_{i}$ of sub-population *i*, respectively. As a result, Model (5) can be extended to

(10) $$\begin{array}{cc} s_{i}^{k+1}= & s_{i}^{k}+h(-\beta_{i}\left(a_{i}^{k}+x_{i}^{k}\right)s_{i}^{k}-\left(\mu_{imax}+\left(\mu_{imin}-\mu_{imax}\right)\eta \right)s_{i}^{k}\\ \; & +\frac{1}{N_{i}}\sum_{j\neq i}\left(\mu_{jmax}+\left(\mu_{jmin}-\mu_{jmax}\right)\eta \right)w_{ij}s_{j}^{k}N_{j}),\\ a_{i}^{k+1}= & a_{i}^{k}+h(q\beta_{i}\left(a_{i}^{k}+x_{i}^{k}\right)s_{i}^{k}-(\left(\mu_{imax}+\left(\mu_{imin}-\mu_{imax}\right)\eta \right)+\sigma_{i}\\ & +\kappa_{i})a_{i}^{k}+\frac{1}{N_{i}}\sum_{j\neq i}\left(\mu_{jmax}+\left(\mu_{jmin}-\mu_{jmax}\right)\eta \right)w_{ij}a_{j}^{k}N_{j}),\\ x_{i}^{k+1}= & x_{i}^{k}+h(\left(1-q\right)\beta_{i}\left(a_{i}^{k}+x_{i}^{k}\right)s_{i}^{k}-\left(\left(\mu_{imax}+\left(\mu_{imin}-\mu_{imax}\right)\eta \right)+\gamma_{i}\right)\\ & x_{i}^{k}+\sigma_{i}a_{i}^{k}+\frac{1}{N_{i}}\sum_{j\neq i}\left(\mu_{jmax}+\left(\mu_{jmin}-\mu_{jmax}\right)\eta \right)w_{ij}x_{j}^{k}N_{j}),\\ r_{i}^{k+1}= & r_{i}^{k}+h(\kappa_{i}a_{i}^{k}+\gamma_{i}x_{i}^{k}-\left(\mu_{imax}+\left(\mu_{imin}-\mu_{imax}\right)\eta \right)r_{i}^{k}\\ \; & +\frac{1}{N_{i}}\sum_{j\neq i}\left(\mu_{jmax}+\left(\mu_{jmin}-\mu_{jmax}\right)\eta \right)w_{ij}r_{j}^{k}N_{j}). \end{array}$$

The following assumption is proposed for Model (10) to be well-defined.

**Assumption** **3.** *For all i,j∈[n] such that $\mu_{i}=\mu_{imax}+\left(\mu_{imin}-\mu_{imax}\right)\eta$, one has $0<h\mu_{i}<1$, $0<h\beta_{i}<1$, $0<h\sigma_{i}<1$, $0<h\gamma_{i}<1$, $0<h\kappa_{i}<1$, $0<h(\beta_{i}+\mu_{i})<1$, $0<h(\gamma_{i}+\mu_{i})<1$, $0<h(\sigma_{i}+\kappa_{i}+\mu_{i})<1$ and $0<h\sum_{j\neq i}\mu_{j}w_{ij}<1$.*

Assumption 3 specifies the parameter scope of Model (10). The sampling parameter is small enough relative to the model parameters.

**Proposition** **2.** *Consider Model (10) under Assumption 3. Suppose $s_{i}^{0},a_{i}^{0},x_{i}^{0},r_{i}^{0}\in \left[0,1\right]$ and $s_{i}^{0}+a_{i}^{0}+x_{i}^{0}+r_{i}^{0}=1$ for all i∈[n]. When k≥0, one has $s_{i}^{k},a_{i}^{k},x_{i}^{k},r_{i}^{k}\in \left[0,1\right]$ and $s_{i}^{k}+a_{i}^{k}+x_{i}^{k}+r_{i}^{k}=1$ for all i∈[n].*

Under Assumption 3, when the control strategy is integrated into the model, Theorem 1 still holds.

## 4. Jointly Data-Driven Identification of Epidemic Parameters and Network Topology

In this section, we discuss an estimation framework of the epidemic parameters and network topology of the networked SAIR model based on real data.

This article aims to train the values of parameters $\beta_{i},\;\sigma_{i},\;\gamma_{i}$, and $\kappa_{i}$ and the network topology, $w_{ij}$, using the collected data. To do so, one defines the following matrices:

(11) $$\begin{array}{c} M_{i}^{l}=M_{i}^{r}\left[\begin{array}{c} \beta_{i}\\ \sigma_{i}\\ \gamma_{i}\\ \kappa_{i} \end{array}\right], \end{array}$$

where $M_{i}^{l}={\left[a_{i}^{1}-a_{i}^{0},\cdots ,a_{i}^{T}-a_{i}^{T-1},x_{i}^{1}-x_{i}^{0},\cdots ,x_{i}^{T}-x_{i}^{T-1},r_{i}^{1}-r_{i}^{0},\cdots ,r_{i}^{T}-r_{i}^{T-1}\right]}^{⊤}$. In addition, one has

$$\begin{array}{cc} \; & M_{i}^{r}=\left[\begin{array}{cccc} hqb_{i}^{0} & -ha_{i}^{0} & 0 & -ha_{i}^{0}\\ \vdots & \vdots & \vdots & \vdots \\ hqb_{i}^{T-1} & -ha_{i}^{T-1} & 0 & -ha_{i}^{T-1}\\ h\left(1-q\right)b_{i}^{0} & ha_{i}^{0} & -hx_{i}^{0} & 0\\ \vdots & \vdots & \vdots & \vdots \\ h\left(1-q\right)b_{i}^{T-1} & ha_{i}^{T-1} & -hx_{i}^{T-1} & 0\\ 0 & 0 & hx_{i}^{0} & ha_{i}^{0}\\ \vdots & \vdots & \vdots & \vdots \\ 0 & 0 & hx_{i}^{T-1} & ha_{i}^{T-1} \end{array}\right], \end{array}$$

where $b_{i}^{T-1}=s_{i}^{T-1}\sum_{j\neq i}w_{ij}\left(a_{j}^{T-1}+x_{j}^{T-1}\right)$. Moreover, for all sub-populations, the epidemic parameters matrix is written as

$$\begin{array}{cc} \; & U=\left[\begin{array}{cccc} u_{11} & u_{12} & u_{13} & u_{14}\\ u_{21} & u_{22} & u_{23} & u_{24}\\ \cdots & \cdots & \cdots & \cdots \\ u_{n1} & u_{n2} & u_{n3} & u_{n4} \end{array}\right]=\left[\begin{array}{cccc} \beta_{1} & \sigma_{1} & \gamma_{1} & \kappa_{1}\\ \beta_{2} & \sigma_{2} & \gamma_{2} & \kappa_{2}\\ \cdots & \cdots & \cdots & \cdots \\ \beta_{n} & \sigma_{n} & \gamma_{n} & \kappa_{n} \end{array}\right]. \end{array}$$

Based on real-life epidemic data, one estimates epidemic parameters and network topology by minimizing the bias on the left and right sides of Equation (11). The identification of the epidemic parameters can be formulated as the least absolute shrinkage and selection operator (LASSO) problem as follows:

(12) $$\begin{array}{cc} \; & \underset{u_{i1},\ldots ,u_{i4}}{min}{\left|\left|M_{i}^{l}-M_{i}^{r}\left(\begin{array}{c} u_{i1}\\ u_{i2}\\ u_{i3}\\ u_{i4} \end{array}\right)\right|\right|}_{2}^{2}+\rho_{i}\sum_{j=1}^{4}u_{ij}.\\ \; & s.t.\;0\leq u_{i1},\ldots ,u_{i4}\leq 1. \end{array}$$

To prevent over-fitting, the second term in objective function (12) represents the $l_{1}$-norm. Moreover, parameter $\rho_{i}$ is determined through cross-validation. Algorithms such as the alternating direction method of multipliers (ADMM) and sequential least squares programming (SLSQP) are used to solve the LASSO problem [22,23]. Then, the SLSQP algorithm is adopted due to its superior performance and ability to handle constrained objective functions. In the joint estimation framework, it is assumed that the topological structure is known, and this known information comes from the actual geographical location. Then, we obtain the parameters $\beta_{i},\;\sigma_{i},\;\gamma_{i},$ and $\kappa_{i}$ with prior knowledge of the network topology by solving the objective function (12). Moreover, by combining Equation (11), the objective function for estimating the network topology is obtained, similar to the function (12). Furthermore, the network topology, $w_{ij}$, can be inferred based on the previously trained epidemic parameters, thus updating the topology.

## 5. Numerical Simulation and Further Discussion

This section demonstrates the proposed mathematical model and theoretical analysis via numerical tests.

### 5.1. Joint Data Collection and Preprocessing

This article is focused on studying the transmission of COVID-19. First, considering that vaccination affects the number of infected people, we select the period during which a substantial portion of the population is vaccinated. Vaccination efforts in Germany commenced in December 2020. Up to March 2021, approximately 9 million individuals had received the first dose of the two required vaccines, while 3 million individuals had already received the second dose. The collected epidemic data in Germany spans from 7 April 2021 to 15 July 2021. Subsequently, the local epidemic and population data are gathered. The collected epidemic data used in this study may be smaller in scale than the actual infection data, as there may have been unreported or undetected cases. A data processing technique is implemented to remove outliers in the collected data [16]. For instance, if there is an outlier in sub-population *i* at time *k* = 6, then we replace the epidemic data at time *k* = 6 with half the sum of the data at time *k* = 5 and *k* = 7 within sub-population *i*. Furthermore, the collected data are divided into two parts for training the epidemic parameters and network topology. One aspect of the data is used as the training set, while the remaining data acts as a test set.

### 5.2. Transmission Prediction

**Figure 1:**
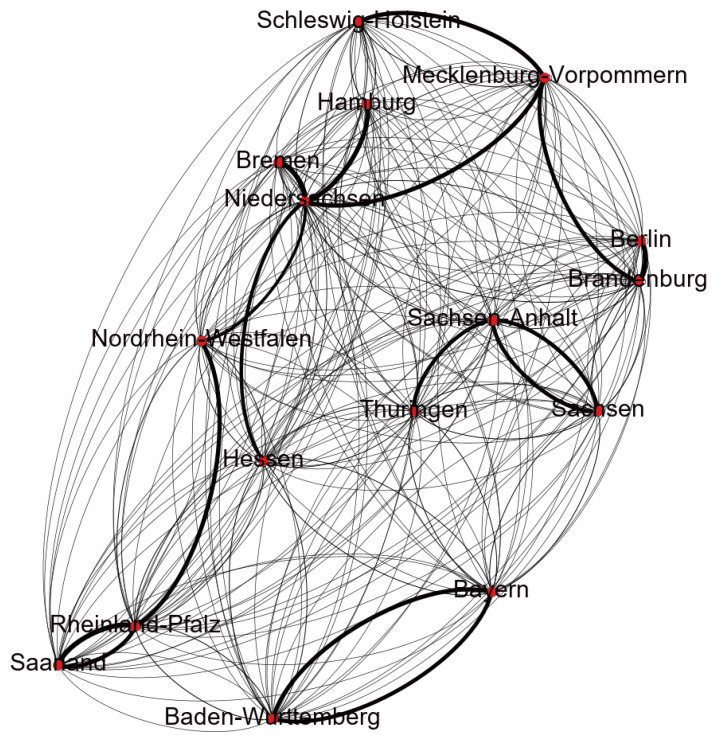
The topology structure of the COVID-19 transmission network in Germany.

**Figure 2:**
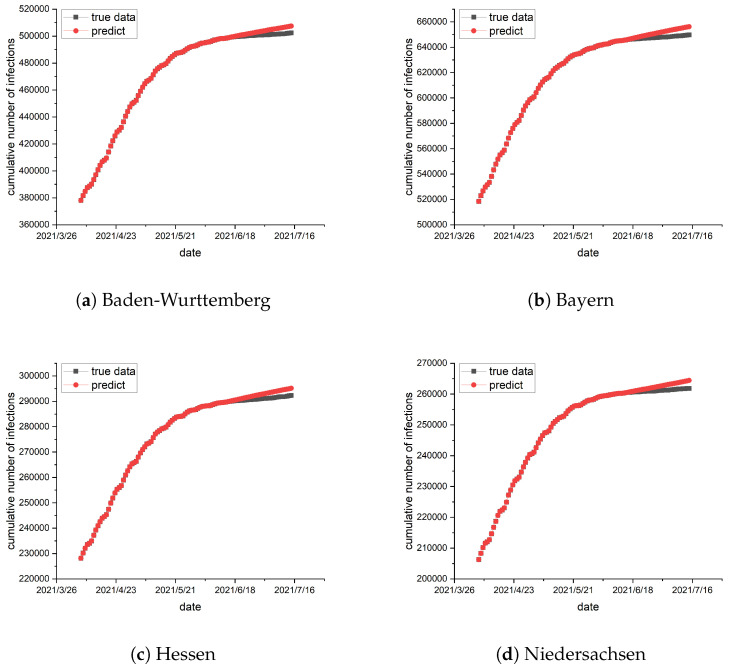
(**a**–**d**) present the simulations conducted in the German states of Baden-Wurttemberg, Bayern, Hessen, and Niedersachsen, respectively. The black curve represents the actual data, while the red represents the simulated values. The predicted results are greater than the real epidemic data, but there is little difference between them. The vertical axis on the left shows the number of infections and the horizontal axis on the bottom shows the date.

**Figure 3:**
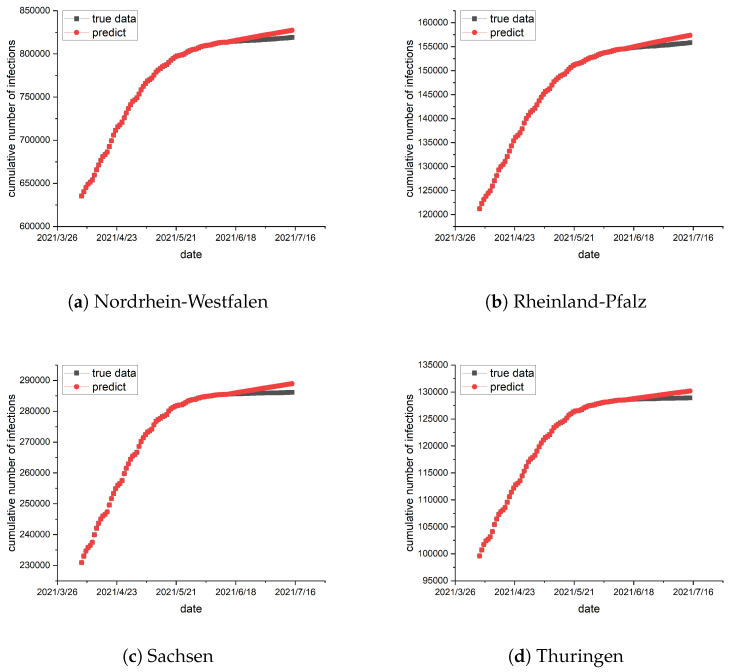
(**a**–**d**) show the simulations in the German states of Nordrhein-Westfalen, Rheinland-Pfalz, Sachsen, and Thuringen, respectively. The black curve represents the actual data, while the red represents the simulated values. The predicted results are greater than the real epidemic data, but there is little difference between them. The vertical axis on the left shows the number of infections and the horizontal axis on the bottom shows the date.

To train the epidemic parameters and network topology, the preceding 70 days of data are utilized as a training set, and the data from the remaining 30 days serve as a test set. The epidemic parameters and network topology are trained using the method described in Section 4. In conjunction with the real geographical location of the German states, the network topology is trained, and the result is exhibited in Figure 1. Subsequently, the trained epidemic parameters and network topology are used for prediction, and the results are illustrated in Figure 2 and Figure 3.

These eight states are used because of their substantial population and many infections. The simulation assumes that the population size is maintained throughout the study period. The points in Figure 1 represent the states of Germany, and the edges represent the interactions between these states. Several significantly larger values are obtained when training the network topology, which are the darker edges in Figure 1. Most of the values are smaller and show little difference, which are the lighter edges in Figure 1. The darker the edge, the greater the interaction intensity between the two states, indicating more frequent traffic between them. Based on the observations from Figure 2 and Figure 3, the results indicate a strong alignment, but there is still some deviation between the predicted and the collected data. The error is always in the same direction, that is, the predicted data is always higher than the actual data. This is because the data and experiments in this article are based on each state. Individual behaviors such as isolation, wearing masks, and social restrictions are difficult to collect and cannot be fully considered in the model, resulting in the predicted infection rate being higher than the actual infection rate. Additionally, we identify similar errors in another reference, suggesting that these errors may come from the modeling process and related data [15]. Moreover, the figures show a consistent increase in the cumulative number of infections, but at a slower pace. The number of susceptible individuals decreases over time. Consequently, with the progress of vaccination, pharmaceutical advancements, and improved immunity, the epidemic is expected to subside eventually. This result illustrates the statement presented in Theorem 1.

**Figure 4:**
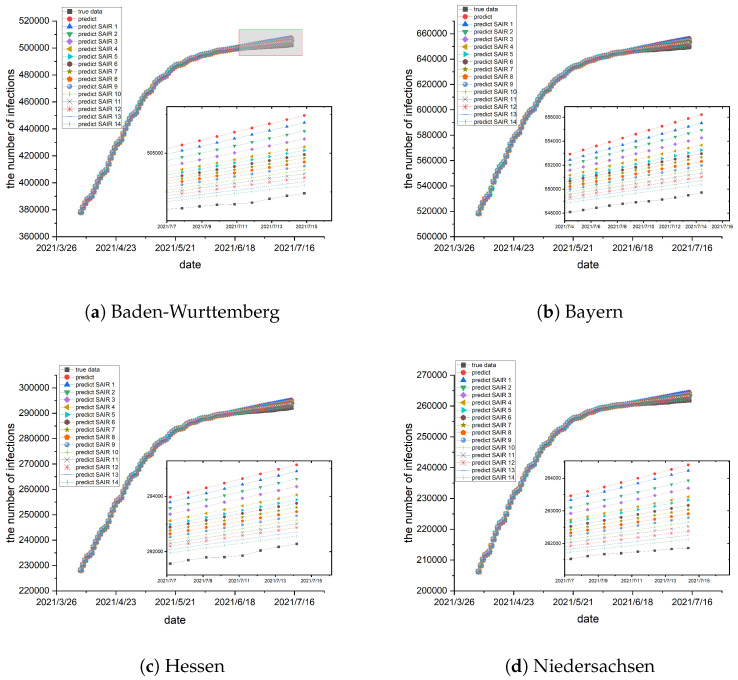
(**a**–**d**) depict the simulations conducted in the German states of Baden-Wurttemberg, Bayern, Hessen, and Niedersachsen, respectively. The black curve represents the real data, while the red dot curve corresponds to the data obtained without using the joint estimation scheme. The remaining curves, characterized by different colors and shapes, represent simulations generated using the cyclic joint estimation scheme. With the cyclic joint estimation scheme, the predicted result curve is gradually closer to the real transmission data, and the error is reduced step by step. The vertical axis on the left shows the number of infections and the horizontal axis on the bottom shows the date.

**Figure 5:**
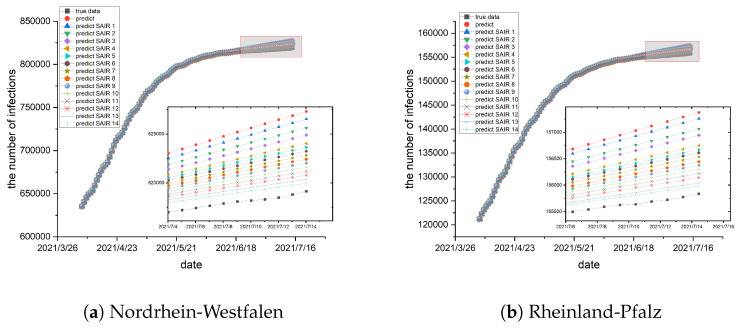

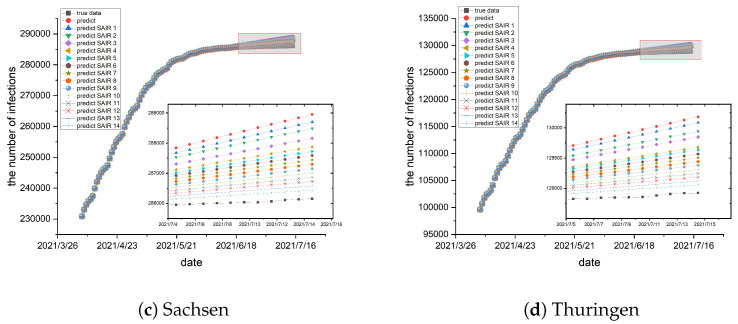
(**a**–**d**) illustrate the simulations conducted in the German states of Nordrhein-Westfalen, Rheinland-Pfalz, Sachsen, and Thuringen, respectively. The black curve represents the real data, while the red dot curve corresponds to the data obtained without using the joint estimation scheme. The remaining curves, characterized by different colors and shapes, represent simulations generated using the cyclic joint estimation scheme. With the cyclic joint estimation scheme, the predicted result curve is gradually closer to the real transmission data, and the error is reduced step by step. The vertical axis on the left shows the number of infections and the horizontal axis on the bottom shows the date.

As described above, a training method for the joint estimation of epidemic parameters and network topology is proposed. Repeated joint estimations are made to enhance the accuracy of the numerical simulations. Initially, we estimate epidemic parameters based on prior knowledge of the network topology, derived from the geographic structure among sub-populations. Subsequently, the network topology is trained and updated using the epidemic parameters from the preceding step. Then, the above two steps are performed in a loop, enhancing the accuracy of the prediction results. The estimation process persists until the simulation error reaches a sufficiently small value, specifically when $\frac{I_{predict}-I_{true}}{I_{true}}\leq 0.1\%$, marking the termination of the estimation process. The simulation results are shown in Figure 4 and Figure 5.

As observed in Figure 4 and Figure 5, the red dotted curve obtained without using the joint estimation scheme is situated at the top of the graph and exhibits the largest deviation from the real data. Subsequently, a simulation using the cyclic joint estimation method is conducted. The initial simulation is denoted by a blue triangular curve labeled “predict SAIR1”, the second simulation is represented by a curve labeled “predict SAIR2”, and so on. The simulation experiment is terminated when the error reaches the required level. The experimental results, presented in Figure 4 and Figure 5, demonstrate that the joint estimation scheme reduces the estimation error and makes the fitting results closer to the real data. In practice, with the ongoing advancement of vaccines and the continuous change of virus structure, the resistance of individuals to viruses is progressively strengthened. Consequently, the probability of infection gradually decreases.

### 5.3. Control Policy

A population flow control strategy, Equation (9), is introduced, in which the mobility rate changes with the change of control intensity. Based on the strategy, one studies the impact of control policies on the spread of epidemics. Moreover, according to official news reports, Germany implemented various measures for epidemic prevention throughout the COVID-19 pandemic. These measures in Germany can be found on Wikipedia, which summarizes the control measures reported by many news and government websites. We collect relevant control policies and divide them into six categories. In the first category, there are restrictions on any non-essential travel. In the second category of control policies, except for food supermarkets, most retail shops need to close. Simultaneously, primary and secondary schools as well as kindergartens must be closed. In the third category, establishments such as restaurants, bars, theaters, and gyms need to close, while schools and regular shops remain unaffected. In category four, stringent measures include the prohibition of groups of gatherings of more than two people. Individuals maintain a 1.5 m distance in public spaces, and the government strictly prohibits activities such as home parties. In the fifth category, public transportation and shops require the wearing of filtering facepiece masks or other clinical-grade masks. In the last category, no control policies are implemented.

The strength of epidemic control measures affects the value of η. To simulate the impact of different policies on disease transmission, several sets of η are defined. For example, case 0-$\eta_{0}$ represents the first set of η, and there are seven sets of η. Each set of η consists of six values, and the value 1 to value 6 corresponds to the six categories described above. For example, value 3 represents the strength of the third category of control policy. The following sets of η are considered in Table 1.

The 300 day epidemic data in the German states is collected, spanning from 17 March 2020 to 10 January 2021. The first 230 days of data are selected as the training set, while the remaining 70 days are used as the test set. The previous survey found that six categories of control policies emerged during the 300-day period. Four categories of control policies emerged in the last 70 days. However, control policies do not necessarily follow the order of the six categories of control policies outlined earlier. To align with the chronological order in which the control policies were implemented during the test set period, the sets of η are reorganized and denoted as $\eta^{\prime }$. The result is shown in Table 2.

**Figure 6:**
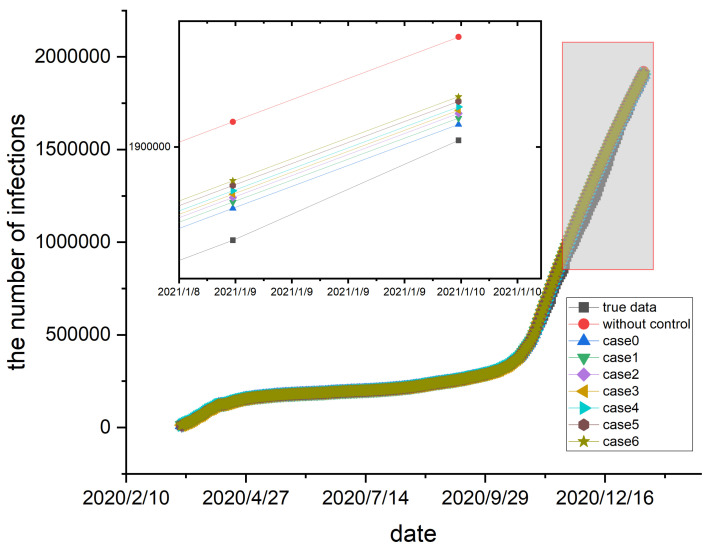
The black curve represents the true data in Germany, while the red curve represents the simulation data obtained without control strategies. The remaining curves represent the simulation data corresponding to each case in Table 2. The curves of the total number of infected individuals are different, with diverse control policies. The vertical axis on the left shows the number of infections and the horizontal axis on the bottom shows the date.

According to Table 2, four distinct control policies are implemented during the test set period. Then, the test set is divided into four stages according to the time sequence. Different control strategies are implemented for each period, corresponding to the four values in the $\eta^{\prime }$ set in order. The simulation results are presented in Figure 6.

According to the simulation results, the absence of control strategies results in the largest error. With the introduction of control strategies, the seven sets of η described above produced different experimental results, namely the curves corresponding to case 0 to case 6 in Figure 6. The curves with the introduction of control strategies show a decrease in the number of infected people compared to the curve without control strategies. Among the results, the simulation result corresponding to $\eta_{0}=\left[1,0.99,0.95,0.90,0.70,0\right]$ demonstrates the best performance and the smallest number of infected people. This observation illustrates that the population flow control strategy limits outdoor activities, avoids more contact infections, and reduces the number of infections during the epidemic. In addition, different population flow control strategies reduce the number of infections differently. Specifically, the closer to a full lockdown, the more effective it is in curbing the spread of the epidemic. However, it is unrealistic to keep a full lockdown. Like the measures the German government took, one should appropriately adjust the control policies to avoid excessive control.

## 6. Conclusions and Future Works

This article introduces a discrete-time networked SAIR model that considers population transportation on the impact of epidemics. The convergence of the model is analyzed and the influence of population migration on the epidemic diffusion is evaluated using the basic reproduction number. Moreover, we propose a joint estimation scheme for epidemic parameters and network topology. Through the simulation experiment, it is found that the application of the joint estimation method greatly reduces the prediction error. In addition, a non-pharmaceutical control strategy is introduced to assess the impact of control policies on epidemic transmission. The experimental results demonstrate that implementing the population flow control strategies proposed in this article can effectively reduce the number of infections during an epidemic. The model is simplified in this work, so the established SAIR model cannot capture the complex transmission dynamics. Moreover, local control is not allowed in this article. Future works include building more complex models, expanding local control to the control strategy, and extending the epidemic model to incorporate a dynamic network structure, then employing model predictive control techniques to regulate the transmission process.

## Figures and Tables

**Table 1:** Sets of η.

Case	Value 1	Value 2	Value 3	Value 4	Value 5	Value 6
case 0-$\eta_{0}$	1	0.99	0.95	0.90	0.70	0
case 1-$\eta_{1}$	1	0.95	0.90	0.75	0.60	0
case 2-$\eta_{2}$	1	0.90	0.85	0.70	0.55	0
case 3-$\eta_{3}$	1	0.90	0.80	0.65	0.50	0
case 4-$\eta_{4}$	1	0.85	0.75	0.70	0.45	0
case 5-$\eta_{5}$	1	0.80	0.70	0.60	0.40	0
case 6-$\eta_{6}$	1	0.75	0.65	0.55	0.30	0

**Table 2:** Sets of $\eta^{\prime }$.

Case	Value 3	Value 4	Value 5	Value 2
case 0-$\eta_{0}^{\prime }$	0.95	0.90	0.70	0.99
case 1-$\eta_{1}^{\prime }$	0.90	0.75	0.60	0.95
case 2-$\eta_{2}^{\prime }$	0.85	0.70	0.55	0.90
case 3-$\eta_{3}^{\prime }$	0.80	0.65	0.50	0.90
case 4-$\eta_{4}^{\prime }$	0.75	0.70	0.45	0.85
case 5-$\eta_{5}^{\prime }$	0.70	0.60	0.40	0.80
case 6-$\eta_{6}^{\prime }$	0.65	0.55	0.30	0.75

## Data Availability

The data set comes from the website at https://github.com/jgehrcke/covid-19-germany-gae/tree/master (accessed on 22 May 2022). The German measures come from the website at https://en.wikipedia.org/wiki/COVID-19_pandemic_in_Germany (accessed on 2 August 2022).

## Appendix A

### Appendix A.1. Proof of Proposition 1

According to the model (5), one has that

$$s_{i}^{k+1}=s_{i}^{k}+h\left(-\beta_{i}\left(a_{i}^{k}+x_{i}^{k}\right)s_{i}^{k}-\frac{1}{n}\sum_{j\neq i}\frac{F_{ji}s_{i}^{k}}{w_{ji}N_{i}}+\frac{1}{N_{i}}\sum_{j\neq i}F_{ij}s_{j}^{k}\right),$$

Since $0<w_{ij}<1$, there exists $\frac{1}{n}\sum_{j\neq i}\frac{F_{ji}s_{i}^{k}}{w_{ji}}>\sum_{j\neq i}F_{ij}s_{j}^{k}$, which yields

$$h\left(-\beta_{i}\left(a_{i}^{k}+x_{i}^{k}\right)s_{i}^{k}-\frac{1}{n}\sum_{j\neq i}\frac{F_{ji}s_{i}^{k}}{w_{ji}N_{i}}+\frac{1}{N_{i}}\sum_{j\neq i}F_{ij}s_{j}^{k}\right)\leq 0,$$

Hence, one can obtain that $s_{i}^{k+1}\leq s_{i}^{k}\leq 1$. Considering the formula $1-h\beta_{i}-h\mu_{i}\leq 1-h\beta_{i}\left(a_{i}^{k}+x_{i}^{k}\right)-h\mu_{i}$ under Assumption 2, there exists the condition $1-h\beta_{i}\left(a_{i}^{k}+x_{i}^{k}\right)-h\mu_{i}\geq 0$. Combined with the model (5), it is noted that $s_{i}^{k+1}\geq 0$. As a result, it holds that $0\leq s_{i}^{k+1}\leq s_{i}^{k}\leq 1$. Additionally, by model (5), one can know that

(A1) $$a_{i}^{k+1}=a_{i}^{k}+h\left(q\beta_{i}\left(a_{i}^{k}+x_{i}^{k}\right)s_{i}^{k}-\left(\sigma_{i}+\kappa_{i}\right)a_{i}^{k}-\iota_{i}^{a}\right),$$

where

$$\iota_{i}^{a}=\mu_{i}a_{i}^{k}-\frac{1}{N_{i}}\sum_{j\neq i}\mu_{j}w_{ij}a_{j}^{k}N_{j}=\frac{1}{n}\sum_{j\neq i}\frac{F_{ji}a_{i}^{k}}{w_{ji}N_{i}}-\frac{1}{N_{i}}\sum_{j\neq i}F_{ij}a_{j}^{k}\geq 0,$$

Thus one gets the inequality as

$$a_{i}^{k+1}\leq a_{i}^{k}+h\left(q\beta_{i}\left(a_{i}^{k}+x_{i}^{k}\right)s_{i}^{k}\right)\leq a_{i}^{k}+s_{i}^{k}\leq 1,$$

Combing with Equation (A1) and Assumption 2, we can obtain that $a_{i}^{k+1}\geq a_{i}^{k}+h\left(-\left(\sigma_{i}+\kappa_{i}+\mu_{i}\right)a_{i}^{k}\right)\geq 0.$ Consequently, the above process shows $0\leq a_{i}^{k+1}\leq 1$. Furthermore, according to model (5), it is worth noting that

(A2) $$x_{i}^{k+1}=x_{i}^{k}+h\left(\left(1-q\right)\beta_{i}\left(a_{i}^{k}+x_{i}^{k}\right)s_{i}^{k}-\gamma_{i}x_{i}^{k}+\sigma_{i}a_{i}^{k}-\iota_{i}^{x}\right),$$

where

$$\iota_{i}^{x}=\mu_{i}x_{i}^{k}-\frac{1}{N_{i}}\sum_{j\neq i}\mu_{j}w_{ij}x_{j}^{k}N_{j}=\frac{1}{n}\sum_{j\neq i}\frac{F_{ji}x_{i}^{k}}{w_{ji}N_{i}}-\frac{1}{N_{i}}\sum_{j\neq i}F_{ij}x_{j}^{k}\geq 0,$$

Thus one gets the inequality as

$$x_{i}^{k+1}\leq x_{i}^{k}+h\left(\left(1-q\right)\beta_{i}\left(a_{i}^{k}+x_{i}^{k}\right)s_{i}^{k}+\sigma_{i}a_{i}^{k}\right)\leq x_{i}^{k}+a_{i}^{k}+s_{i}^{k}\leq 1,$$

Combing with Equation (A2) and Assumption 2, there exists $x_{i}^{k+1}\geq x_{i}^{k}+h\left(-\left(\gamma_{i}+\mu_{i}\right)x_{i}^{k}\right)\geq 0$, which yields $0\leq x_{i}^{k+1}\leq 1$. Moreover, by model (5), one can obtain that

(A3) $$r_{i}^{k+1}=r_{i}^{k}+h\left(\kappa_{i}a_{i}^{k}+\gamma_{i}x_{i}^{k}-\iota_{i}^{r}\right),$$

where

$$\iota_{i}^{r}=\mu_{i}r_{i}^{k}-\frac{1}{N_{i}}\sum_{j\neq i}\mu_{j}w_{ij}r_{j}^{k}N_{j}=\frac{1}{n}\sum_{j\neq i}\frac{F_{ji}r_{i}^{k}}{w_{ji}N_{i}}-\frac{1}{N_{i}}\sum_{j\neq i}F_{ij}r_{j}^{k}\geq 0,$$

Thus one gets the inequality as

$$r_{i}^{k+1}\leq r_{i}^{k}+h\left(\kappa_{i}a_{i}^{k}+\gamma_{i}x_{i}^{k}\right)\leq a_{i}^{k}+x_{i}^{k}+r_{i}^{k}\leq 1,$$

Combing with Equation (A3) and Assumption 2, there exists $r_{i}^{k+1}\geq \left(1-h\mu_{i}\right)r_{i}^{k}\geq 0.$ Hence, one can get the result $0\leq r_{i}^{k+1}\leq 1$. Besides, at the moment *k*, it follows that $S_{i}^{k}+A_{i}^{k}+X_{i}^{k}+R_{i}^{k}=N_{i}^{k}$. At the moment k+1, there exists $S_{i}^{k+1}+A_{i}^{k+1}+X_{i}^{k+1}+R_{i}^{k+1}=N_{i}^{k+1}$. With Assumption 2, it holds that $N_{i}^{k}=N_{i}^{k+1}$. Thanks to $s_{i}^{k}+a_{i}^{k}+x_{i}^{k}+r_{i}^{k}=\frac{S_{i}^{k}+A_{i}^{k}+X_{i}^{k}+R_{i}^{k}}{N_{i}^{k}}=1$, one has $s_{i}^{0}+a_{i}^{0}+x_{i}^{0}+r_{i}^{0}=s_{i}^{k}+a_{i}^{k}+x_{i}^{k}+r_{i}^{k}=s_{i}^{k+1}+a_{i}^{k+1}+x_{i}^{k+1}+r_{i}^{k+1}=1$.

### Appendix A.2. Proof of Theorem 1

The above proof of Proposition 1 shows that $s_{i}^{k}$ is monotonous and non-increasing, which yields $s_{i}^{k+1}\leq s_{i}^{k}$. Using $S^{k}=\sum_{i=1}^{n}s_{i}^{k}$, $A^{k}=\sum_{i=1}^{n}a_{i}^{k}$, $X^{k}=\sum_{i=1}^{n}x_{i}^{k}$ for all i∈[n] to indicate the sum of susceptible, asymptomatic and infected states of all sub-populations, the model (5) thus turns into

$$\begin{array}{ccc} \; & S^{k+1}=S^{k}-h\sum_{i=1}^{n}\beta_{i}\left(a_{i}^{k}+x_{i}^{k}\right)s_{i}^{k}, & \\ \; & A^{k+1}=A^{k}+h\left(q\sum_{i=1}^{n}\beta_{i}\left(a_{i}^{k}+x_{i}^{k}\right)s_{i}^{k}-\sum_{i-1}^{n}\left(\sigma_{i}+\kappa_{i}\right)a_{i}^{k}\right), & \\ \; & X^{k+1}=X^{k}+h\left(\left(1-q\right)\sum_{i=1}^{n}\beta_{i}\left(a_{i}^{k}+x_{i}^{k}\right)s_{i}^{k}+\sum_{i=1}^{n}\sigma_{i}a_{i}^{k}-\sum_{i=1}^{n}\gamma_{i}x_{i}^{k}\right), \end{array}$$

Under Assumption 2 and Proposition 1, it is known that $s_{i}^{k}$ is non-increasing, so the lower bound for $s_{i}^{k}$ is 0. Considering the condition $\lim_{k\to \infty }S^{k+1}-S^{k}=-h\sum_{i=1}^{n}\beta_{i}\left(a_{i}^{k}+x_{i}^{k}\right)s_{i}^{k}=0$, we obtain

$$\lim_{k\to \infty }-hq\sum_{i=1}^{n}\beta_{i}\left(a_{i}^{k}+x_{i}^{k}\right)s_{i}^{k}=\lim_{k\to \infty }-h\left(1-q\right)\sum_{i=1}^{n}\beta_{i}\left(a_{i}^{k}+x_{i}^{k}\right)s_{i}^{k}=0,$$

Therefore, one can get $\lim_{k\to \infty }A^{k+1}-A^{k}=-h\sum_{i=1}^{n}\left(\sigma_{i}+\kappa_{i}\right)a_{i}^{k}=0$. By Assumption 2, it is noted that $0<h(\sigma_{i}+\kappa_{i})<1$, which leads to $\lim_{k\to \infty }a_{i}^{k}=0$. Similarly, it can also be proved that $\lim_{k\to \infty }x_{i}^{k}=0$.

The proof of the third conclusion in Theorem 1 is as follows. Based on Assumption 2, it is evident that each element of matrix $M_{k}$ is non-negative, and the matrix is irreducible. According to the Perron-Frobenius theorem for non-negative irreducible matrices, it is known that $\lambda_{max}^{M_{k}}=\rho \left(M_{k}\right)$. The theorem further asserts that any decrease in the elements of $M_{k}$ leads to a corresponding decrease in $\rho (M_{k})$. If $s_{i}^{k+1}<s_{i}^{k}$, $\rho \left(M_{k}\right)>\rho \left(M_{k+1}\right)$, which implies $\lambda_{max}^{M_{k}}>\lambda_{max}^{M_{k+1}}$.

The proof of the last conclusion is also provided. If a moment $k_{0}$ is identified at which the model converges to equilibrium, one has $\lambda_{max}^{M_{k}}<1$. Therefore, there exists a specific $k_{0}$ such that $\lambda_{max}^{M_{k}}<1$ for all $k\geq k_{0}$. There are two scenarios for the proof. Firstly, if $\lim_{k\to \infty }s^{k}=0$, one can have

(A4) $$\begin{array}{c} M_{k}=\left[\begin{array}{cc} I+h\left(-\left(\sigma +\kappa +\mu \right)+N^{-1}W\mu N\right) & 0\\ h\sigma & I+h\left(-\left(\gamma +\mu \right)+N^{-1}W\mu N\right) \end{array}\right]. \end{array}$$

According to Equation (A4), the diagonal term of the matrix is between 0 and 1. Thus there exists $k_{0}$ such that $\lambda_{max}^{M_{k}}<1$ for $k\geq k_{0}$. Secondly, if $\lim_{k\to \infty }s^{k}=s^{*}\neq 0_{n}$, the equilibrium form to which the system converges is $\left(s^{*},0,0,1-s^{*}\right)$. By defining $\varphi_{s}^{k}=s^{k}-s^{*}$, $\varphi_{p}^{k}=p^{k}-0_{n}$, one has that $\lim_{k\to \infty }\varphi_{s}^{k}=\varphi_{p}^{k}=0_{n},$ where $p^{k}={\left[a^{k},x^{k}\right]}^{Τ}$. Then, we move the item to get that

(A5) $$\begin{array}{cc} \; & \varphi_{s}^{k+1}-\varphi_{s}^{k}=s^{k+1}-s^{k}=-hB\mathrm{diag}\left(s_{i}^{k}\right)\left(a^{k}+x^{k}\right),\\ \; & \varphi_{p}^{k+1}=M_{k}\varphi_{p}^{k}, \end{array}$$

where $\lambda_{max}^{M_{k}}$ is the dominant eigenvalue of matrix $M_{k}$ and there is a corresponding eigenvector v, then there exists $v^{Τ}M_{k}=\lambda_{max}^{M_{k}}v^{Τ}$. According to Equation (A5), if $\lambda_{max}^{M_{k}}>1$, one has that $\varphi_{p}^{k+1}>\varphi_{p}^{k}$, which contradicts the condition that the system reaches equilibrium. On the other hand, if $\lambda_{max}^{M_{k}}=1$, it implies $v^{Τ}M_{k}=v^{Τ}$, which means that matrix $M_{k}$ must be an identity matrix, leading to the contradiction that $\lim_{k\to \infty }s^{k}=s^{*}=0_{n}$. Thus, when the model converges to a stable equilibrium at the instant $k_{0}$, it holds that $\lambda_{max}^{M_{k}}<1$. Consequently, there exists $k_{0}$ such that $\lambda_{max}^{M_{k}}<1$ for $k\geq k_{0}$.
